# Miscarriage risk assessment: a bioinformatic approach to identifying candidate lethal genes and variants

**DOI:** 10.1007/s00439-023-02637-y

**Published:** 2024-02-01

**Authors:** Mona Aminbeidokhti, Jia-Hua Qu, Shweta Belur, Hakan Cakmak, Eleni Jaswa, Ruth B. Lathi, Marina Sirota, Michael P. Snyder, Svetlana A. Yatsenko, Aleksandar Rajkovic

**Affiliations:** 1 Department of Pathology, University of California San Francisco, San Francisco, CA 94143 USA; 2 Division of Reproductive Endocrinology and Infertility, Department of Obstetrics, Gynecology, and Reproductive Sciences, University of California, San Francisco, CA 94143 USA; 3 Department of Obstetrics, Gynecology and Reproductive Sciences, University of California San Francisco, San Francisco, CA 94143 USA; 4 Division of Reproductive Endocrinology and Infertility, Department of Obstetrics and Gynecology, Stanford University, Stanford, CA 94305 USA; 5 Department of Pediatrics, University of California, San Francisco, San Francisco, CA 94143 USA; 6 Bakar Computational Health Sciences Institute, University of California, San Francisco, San Francisco, CA 94143 USA; 7 Department of Genetics, School of Medicine, Stanford University, Stanford, CA 94305 USA; 8 Department of Pathology, University of Pittsburgh, Pittsburgh, PA 15213 USA; 9 Department of Obstetrics, Gynecology and Reproductive Sciences, University of Pittsburgh, Pittsburgh, PA 15213 USA; 10 Magee-Women Research Institute, Pittsburgh, PA 15213 USA; 11 Institute of Human Genetics, University of California San Francisco, San Francisco, CA 94143 USA

## Abstract

**Purpose:**

Miscarriage, often resulting from a variety of genetic factors, is a common pregnancy outcome. Preconception genetic carrier screening (PGCS) identifies at-risk partners for newborn genetic disorders; however, PGCS panels currently lack miscarriage-related genes. In this study, we evaluated the potential impact of both known and candidate genes on prenatal lethality and the effectiveness of PGCS in diverse populations.

**Methods:**

We analyzed 125,748 human exome sequences and mouse and human gene function databases. Our goals were to identify genes crucial for human fetal survival (lethal genes), to find variants not present in a homozygous state in healthy humans, and to estimate carrier rates of known and candidate lethal genes in various populations and ethnic groups.

**Results:**

This study identified 138 genes in which heterozygous lethal variants are present in the general population with a frequency of 0.5% or greater. Screening for these 138 genes could identify 4.6% (in the Finnish population) to 39.8% (in the East Asian population) of couples at risk of miscarriage. This explains the cause of pregnancy loss in approximately 1.1–10% of cases affected by biallelic lethal variants.

**Conclusion:**

This study has identified a set of genes and variants potentially associated with lethality across different ethnic backgrounds. The variation of these genes across ethnic groups underscores the need for a comprehensive, pan-ethnic PGCS panel that includes genes related to miscarriage.

**Supplementary Information:**

The online version contains supplementary material available at 10.1007/s00439-023-02637-y.

## Introduction

Human reproduction is inefficient, with ~70% of all conceptions not progressing to live birth across different developmental stages (Larsen et al. 2013; Tise and Byers 2021). Reproductive failures often happen in the early stages after conception, where approximately 10–30% of embryos stop developing before implantation (Larsen et al. 2013; Montjean et al. 2021; Tise and Byers 2021). An additional 30% of losses occur after implantation, within 3–4 weeks of gestation, before the pregnancy is even recognized (Larsen et al. 2013). Finally, 10–20% of clinically recognized pregnancies are lost in the first trimester (Larsen et al. 2013). Non-viable conceptions show a range of outcomes. Lethality can occur at various stages of fetal development, leading to outcomes such as early pregnancy loss, spontaneous abortion, intrauterine fetal demise, stillbirth, or death in neonatal or early childhood stages.

Fetal chromosomal abnormalities are associated with 50% of first-trimester miscarriages, while the etiology of pregnancy loss in the remaining 50% of cases with normal karyotype and chromosomal microarray analysis remains unexplained (Hardy and Hardy 2018). Recent exome sequencing studies have begun to uncover deleterious variants in specific genes that cause euploid pregnancy loss (Najafi et al. 2021; Rajcan-Separovic 2020; Robbins et al. 2019; Workalemahu et al. 2023; Xiang et al. 2021; Zhao et al. 2021). However, the spectrum of genes associated with human lethality and pregnancy loss remains understudied.

Identifying genes linked to pregnancy losses in humans is challenging. This is mainly due to the lack of large-scale studies that assess pregnancy losses from fertilization to clinical presentation and appropriately categorize these losses. Moreover, damaging variants in some critical genes may affect early stages of development and not be associated with any particular phenotype archived in the Online Mendelian Inheritance in Man [OMIM] database.

Out of ~20,000 human genes, only a quarter is associated with any known phenotype, the majority of which are observed postnatally (Dawes et al. 2019). Based on current OMIM entries, only 624 (3.7%) human genes are linked to lethal phenotypes that can lead to fetal, perinatal, or childhood mortality (Dawes et al. 2019). The survival duration of patients with pathogenic variants in these genes can vary based on the severity of the disease. The actual set of human genes essential to maintain fetal viability may be much larger as 39.2% of mouse genes are associated with lethality through knockouts (Dawes et al. 2019; Dickinson et al. 2016). Previous studies have estimated that ~3,400 of human genes are essential for embryonic and fetal survival (Dawes et al. 2019). The potential contribution of candidate genes to human lethal phenotypes and pregnancy losses is unknown.

The objective of this study was to use existing human exome datasets and discern genes that could be potentially added to PGCS to identify couples at risk for pregnancy loss. We also used a current database of human exomes to evaluate the frequency of pathogenic, likely pathogenic, and putative loss-of-function variants in targeted genes (Gudmundsson et al. 2022; Guo and Gregg 2019). We calculated the distribution of such variants across different ancestral populations and the probability that such variants contribute to human pregnancy losses. We identified 138 known and candidate lethal genes that have a high-frequency rate of ≥0.5% (1/200 individuals) of potentially damaging variants and are present among different ethnic groups. These 138 genes may significantly contribute to pregnancy losses and could be included in PGCS. This would enable preimplantation genetic testing to prevent pregnancy loss in at-risk couples. Future studies will be needed to determine the validity of our conclusions.

## Materials and methods

### Selection of lethal candidate genes and variant filtration

**Fig. 1:**
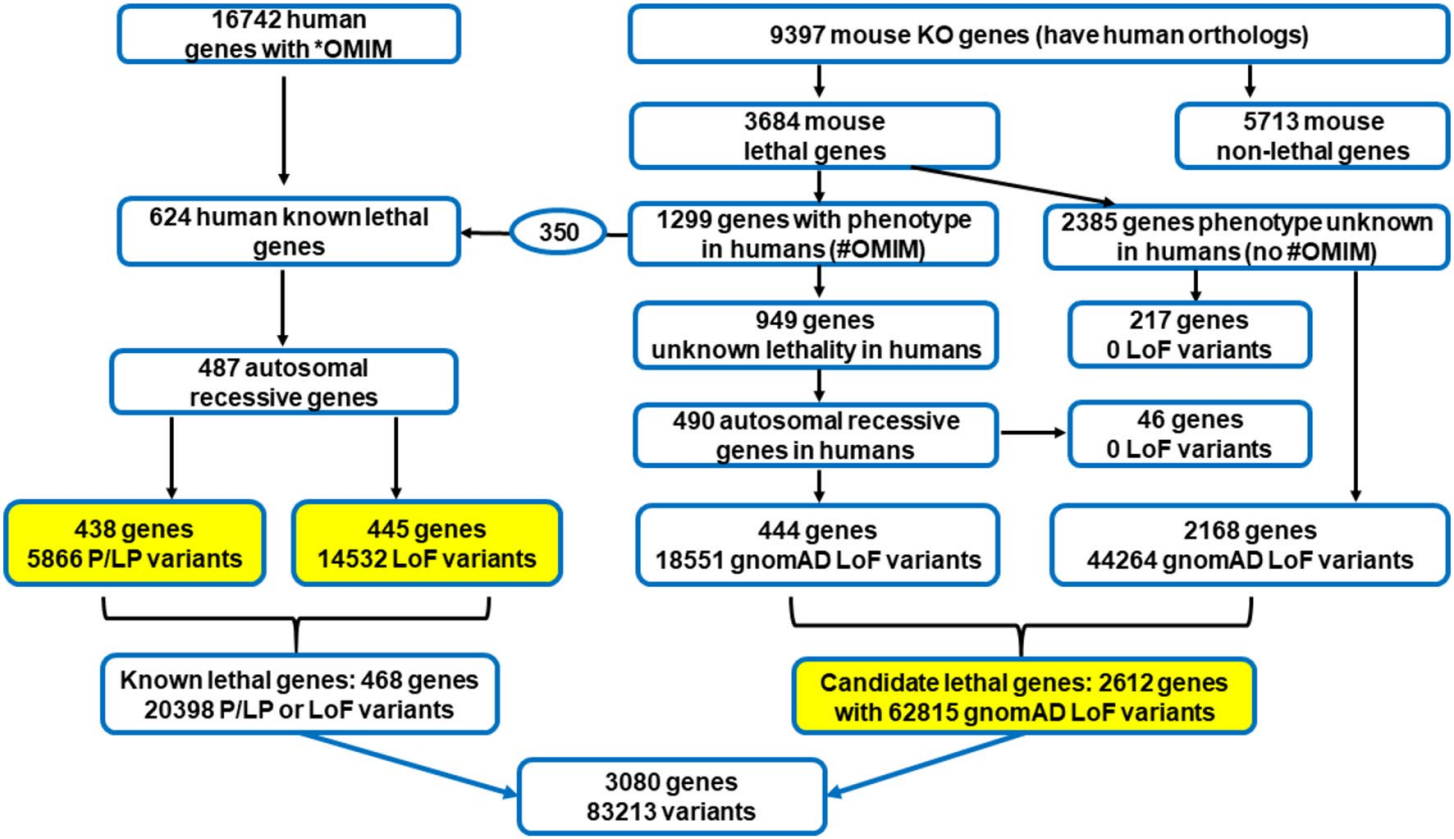
Bioinformatic Workflow for Identifying Genes Associated with Human Lethality. This figure illustrates a bioinformatic pipeline used to delineate known and candidate genes implicated in human lethality. Starting on the left, the Online Mendelian Inheritance in Man (OMIM) database was queried to isolate genes implicated in human lethality, yielding 624 known lethal human genes. From these, genes with autosomal recessive patterns were isolated for an in-depth analysis of their associated pathogenic/likely pathogenic (P/LP) and loss-of-function (LoF) variants, excluding those already classified as P/LP. Moving to the right side of the workflow, we identified candidate genes (potentially lethal in humans) informed by lethal phenotypes observed in mouse knock-out (KO) studies. Within the OMIM database, 1,299 of the 3,684 genes had documented human phenotypes, including 350 genes previously recognized for their association with prenatal lethality. These 350 genes were thus excluded from the candidate gene set, as they were already accounted for among the known lethal genes. The remaining 949 genes have human phenotypes documented in OMIM but have not yet been reported as lethal. Out of these, 490 genes demonstrating recessive inheritance were earmarked for additional investigation. The other 2,385 genes out of the 3,684 total did not have OMIM entries, lacked associated clinical phenotypes, or had undefined modes of inheritance. In total, 2,875 (490 + 2,385) candidate genes underwent population-based analysis and a search for LoF variants in the gnomAD database, which led to findings on 2,612 genes. The cumulative count of genes along with their P/LP and LoF variants are displayed at the bottom of the figure. Highlighted in yellow are three datasets that were analyzed

In this study, we investigated genes related to human lethality, including “known lethal genes” and “candidate lethal genes”. Among nuclear genes (16,742) deposited in OMIM, 6,406 genes had an associated human phenotype. Of these, 624 genes (Dawes et al. 2019) were documented as having perinatal lethal human phenotypes, including 120 with autosomal dominant, 17 with X-linked, and 487 with autosomal recessive inheritance. For the purpose of this study, we focused on 487 genes with autosomal recessive inheritance, defined as “known lethal genes” (Fig. 1). ClinVar curated pathogenic [P] and likely pathogenic [LP] variants were downloaded from the Genome Aggregation Database [gnomAD] v2.1.1 (Karczewski et al. 2020). In addition, loss-of-function [LoF] variants including nonsense, frameshift, insertions/deletions, and splice site alterations that disrupt the reading frame of protein-coding genes, were obtained from gnomAD and included in the analysis. For each variant, the extracted parameters contained allele frequencies for the entire population acquired from 125,748 exome sequences, as well as for available ethnic groups: African/African American, Ashkenazi Jewish, East Asian, Finnish, Latino/Admixed American, Non-Finnish European, and South Asian (Supplemental data, Table S1).

We also generated a human “candidate lethal gene” list, derived from a set of 9,397 genes with mouse knock-out phenotypes extracted from the Mouse Genome Informatics and International Mouse Phenotyping Consortium databases as previously described (Dawes et al. 2019). Knockouts for 3,684 genes, which had human orthologs, induced pre-weaning lethality in mice. A series of filters (supplemental data) was applied to extract LoF variants observed in a heterozygous but not in a homozygous state in 125,748 exomes of healthy adults (Fig. 1). We assumed that these variants are functional, fully penetrant, and are inherited in an autosomal recessive fashion in humans as in mouse models. We excluded structural, missense, or other types of variants due to the uncertainty of their functional significance in candidate genes.

### Population risk probability metrics

To estimate population risk probabilities, calculations were done on three sets of variants: P/LP variants in the “known lethal genes”, LoF variants in the “known lethal genes”, and LoF variants in the “candidate lethal genes”. The metrics computed included variant carrier rate [VCR]—the frequency of a variant in the general population; gene carrier rate [GCR]—the cumulative frequency of all P/LP or LoF variants in a specific gene; and at-risk couples rate [ACR]—the proportion of couples where both partners carry a potentially lethal variant in the same gene (supplemental data, Guo and Gregg 2019). Calculations were also performed for each ancestry.

### Gene ontology annotations of known and candidate lethal genes

The top genes with GCR ≥ 0.005 were subjected to ontology analyses using the PANTHER (Protein Analysis Through Evolutionary Relationships) Classification System version-17 (http://pantherdb.org). Sixty-one “known lethal genes” and 77 “candidate lethal genes” were annotated according to the Gene Ontology Biological Process, Molecular Function, Cellular Component enrichment, Protein class and implicated Pathways.

## Results

### Lethal gene variants in the general population

In 125,748 exomes of healthy individuals, among 487 “known lethal genes”, P/LP and LoF variants were detected in 438 and 445 genes, respectively. In total, 468 genes had at least one P/LP and/or LoF variant, while 19 genes had neither P/LP nor LoF variants recorded (Fig. 1). In gnomAD, 62,815 LoF variants were annotated for 2,612 genes in the general human population and included in the “candidate lethal genes” analysis. Calculations were made for three sets: 5,866 P/LP variants (all types classified as P/LP by ClinVar) in 438 “known lethal genes”; 14,532 LoF variants (not classified as P/LP by ClinVar) in 445 “known lethal genes”; and 62,815 LoF variants in 2612 “candidate lethal genes” (Fig. 1). Among the “known lethal genes”, 94/5,866 (1.6%) of P/LP and 54/14,532 (0.37%) of LoF variants were seen with a frequency of ≥0.5% or 1/200 individuals in the general population (supplementary Fig. S1A, B). In “candidate lethal genes”, 519/62,815 (0.83%) of LoF variants had VCR** ≥ **0.5%. The remaining LoF variants were rare with VCR < 0.5% in the general population (Supplementary Fig. S1C).

### Gene carrier rates for P/LP and LoF variants in the “known lethal genes”

**Fig. 2:**
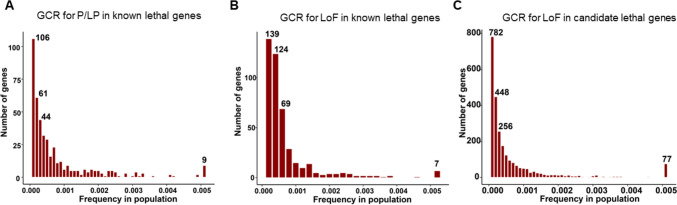
Distribution of Gene Carrier Rates for Pathogenic Variants in the General Population. Panel **A** displays the gene carrier rate (GCR) for pathogenic/likely pathogenic (P/LP) variants within known lethal genes, highlighting that 0.5% of the general population carries a P/LP variant in one of the nine identified lethal genes, corresponding to a frequency of 0.005. Panel **B** details the GCR for loss-of-function (LoF) variants in known lethal genes, with LoF variants present in 0.5% of the population for seven of these genes. Panel **C** illustrates the GCR for LoF variants in candidate lethal genes, showing that LoF variants in 77 candidate lethal genes are found in 0.5% of the general population

A cumulative frequency of variants in a given gene (GCR) was calculated for P/LP variants in “known lethal genes”, LoF variants in “known lethal genes”, and LoF variants in “candidate lethal genes”. In the general population, GCR** ≥ **0.5% was identified for 9/438 known genes with P/LP variants (*ABCC6* [MIM *603234]*, DHCR7* [MIM *602858]*, F7* [MIM *613878]*, HBB* [MIM *141900], *KIAA0586* [MIM *610178]*, PKHD1* [MIM *606702]*, PMM2* [MIM *601785], *SBDS* [MIM *607444], *SPINK5* [MIM *605010]), 7/445 known genes with LoF variants (*ABCC6* [MIM *603234]*, CEP290* [MIM *610142], *F7* [MIM *613878]*, KIAA1109* [MIM *611565]*, NPC2* [MIM *601015]*, PKHD1* [MIM *606702]*, SPEG* [MIM *615950]), and 77/2,612 candidate genes with LoF variants (Fig. 2, Table S2).

**Fig. 3:**
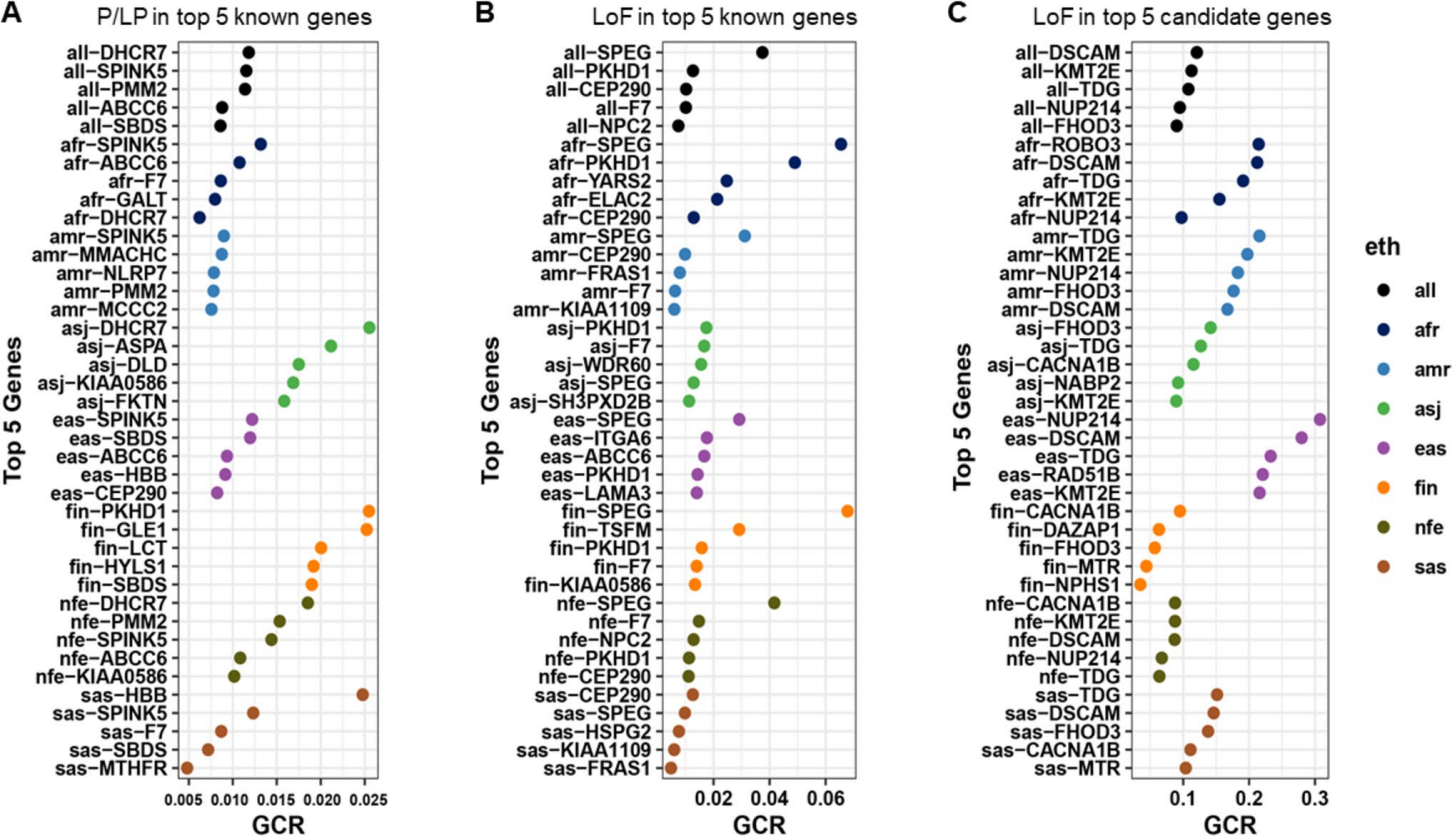
Prevalence of Gene Carrier Rates for Lethal Variants Across Populations. The figure illustrates the top five lethal genes with the highest gene carrier rates (GCR). Panel **A** represents GCR values for pathogenic/likely pathogenic (P/LP) variants in known lethal genes, while panel **B** shows GCR values for loss-of-function (LoF) variants in the same set of genes. Panel **(C)** pertains to LoF variants in candidate lethal genes. The most significant GCR values across the overall population are marked in black. Furthermore, the figure identifies the top five genes within seven ethnic groups: African/African American (afr), Latino/Admixed American (amr), Ashkenazi Jewish (asj), East Asian (eas), Finnish (fin), Non-Finnish European (nfe), and South Asian (sas). Detailed information regarding genes with a GCR of 0.005 or greater, whether in all populations combined or in individual ethnic groups, can be found in Table S2

The five highest GCRs for each gene set in the general population and in each ethnic group are shown in Fig. 3. P/LP and LoF variants in 18 known lethal genes *ABCC6*, *BCKDHB* [MIM *248611], *CEP290*, *DHCR7*, *F7*, *GBA* [MIM *606463], *HBB*, *KIAA0586* [MIM *610178], *KIAA1109* [MIM *611565], *NLRP7* [MIM *609661], *NPC2*, *PKHD1*, *PMM2*, *PYGM* [MIM *608455], *RARS2* [MIM *611524], *SBDS*, *SPEG*, and *SPINK5* [MIM *605010] were present with GCR** ≥ **0.5% in two or more ethnic groups (Fig. 3). Each population had a unique set of genes, variants in which are more prevalent in individuals of that ethnicity (Supplementary Fig. S2, Table S2). Among P/LP variants, the highest rates were observed for the *DHCR7* (GCR = 0.026) and *ASPA* [MIM *608034] (GCR = 0.021) genes in Ashkenazi Jewish population, for the *PKHD1* (GCR = 0.025) and *GLE1* [MIM *603371] (GCR = 0.025) genes in Finnish population, and for the *HBB* gene (GCR = 0.025) in South Asians (Fig. 3A).

Among LoF variants*, SPEG*, a known lethal gene, had the highest GCR in multiple ethnicities (Fig. 3B). LoF variants in the *ELAC2* (MIM *605,367) (GCR = 0.021), *PKHD1*(GCR = 0.049), and *YARS2* [MIM *610957] (GCR = 0.025) genes were the most frequent in African/African American population, and LoF variants in the *TSFM* gene [MIM *604723] (GCR = 0.029) were common in Finnish population (Fig. 3B). Table S2 provides a list of 43 known lethal genes that are common (GCR** ≥ **0.5%) among individuals of a specific ethnicity.

### Gene carrier rates for LoF variants in the “candidate lethal genes”

Top ten genes with the GCR ≥ 0.05 among the human “candidate lethal genes” were *DSCAM* [MIM *602523] (GCR = 0.121), *KMT2E* [MIM *608444] (GCR = 0.113), *TDG* [MIM *601423] (GCR = 0.108), *NUP214* [MIM *114350] (GCR = 0.095), *FHOD3* [MIM *609691] (GCR = 0.090), *CACNA1B* [MIM *601012] (GCR = 0.081), *NABP2* [MIM *612104] (GCR = 0.069), *MTR* [MIM *156570] (GCR = 0.068), *RAD51B* [MIM *602948] (GCR = 0.060), and *ABCF1* [MIM *603429](GCR = 0.045) (Table S2). Among the candidate lethal genes, *NUP214* and *DSCAM* genes had the highest GCR for LoF variants in the East Asian ethnic group (Fig. 3C) and were also present in the top genes in other populations. Candidate lethal genes with GCR ≥ 0.5% in the general population are listed in the Table S2.

### At-risk couple rates in the general and distinct ethnic populations

**Fig. 4:**
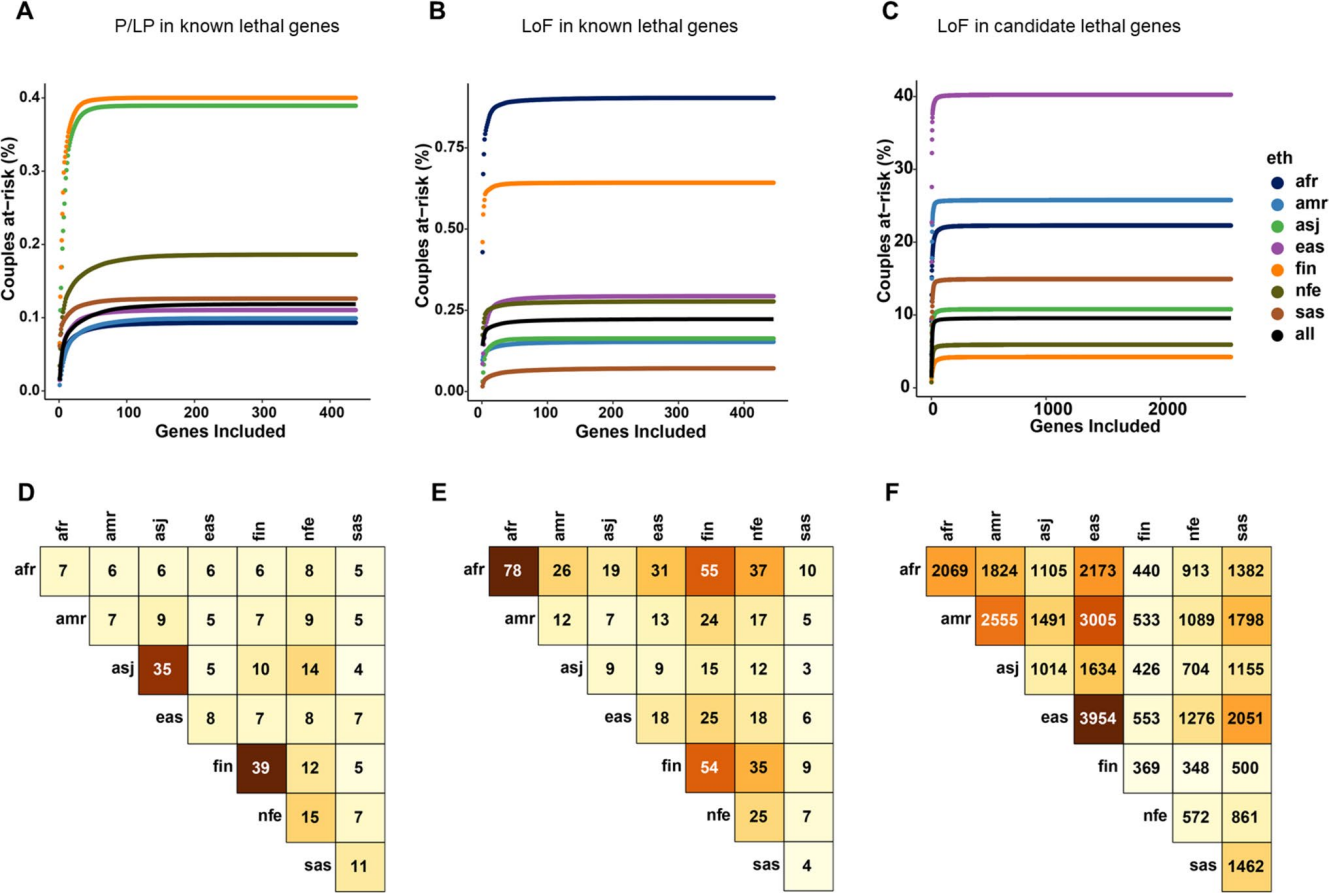
At-risk couple rates (ACRs). This figure presents the cumulative probability curves for couples at genetic risk within various populations based on **A** pathogenic/likely pathogenic (P/LP) variants and **B** loss-of-function (LoF) variants in known lethal genes. Panel **C** illustrates the cumulative risk for couples concerning LoF variants in candidate lethal genes across ethnic groups: African/African American (afr), Latino/Admixed American (amr), Ashkenazi Jewish (asj), East Asian (eas), Finnish (fin), Non-Finnish European (nfe), and South Asian (sas). The general population curve is accentuated in black. Panels **D–F** detail the rates of at-risk couples within and between ethnicities, calculated for 138 genes. The at-risk couple rates (ACRs) for intra-ancestry couples, those from the same ancestry, and inter-ancestry couples, those from different ancestries, are shown as the number of couples per 10,000. Panel **D** shows the number of couples at risk of conceiving an embryo with two P/LP variants, and panel **E** with two LoF variants, for genes on the list of known lethal genes. Panel **F** presents the number of couples at risk of having a conception with two inherited LoF variants in a candidate lethal gene

At-risk couple rates [ACRs] were calculated for P/LP variants in “known lethal genes”, LoF variants in “known lethal genes”, and LoF variants in “candidate lethal genes”. Cumulative curves for all genes are shown in Fig. 4A–C. Up to 0.4% of couples are at risk of having a pregnancy/child affected by one of the autosomal recessive conditions associated with lethal phenotype due to the inheritance of P/LP variants in human “known lethal genes” (Fig. 4A). If LoF variants in the known and candidate genes are included, an additional ~ 0.2% (Fig. 4B) and 9.8% (Fig. 4C) of couples in the general population are at risk of adverse reproductive outcomes. Therefore, in the general population at least 10% of couples who are heterozygous for P/LP and LoF variants in lethal genes may be at-risk of a conception with homozygous/compound heterozygous genotypes and a subsequent pregnancy loss. Moreover, at-risk rate depends on the couple’s ethnicity (Fig. 4). ACR values were also calculated in each distinct human population. The African/African American and Finnish populations have the highest risk for LoF variants in the “known lethal genes” (Fig. 4B). Among the “candidate lethal genes”, the highest ACR value is observed for the East Asian ethnicity (Fig. 4C). Theoretically, 25% of conceptions from at-risk couples will be affected by biallelic lethal variants, leading to a miscarriage or a neonatal death.

Risk values were also calculated for the inter-ancestry and intra-ancestry couples for 138 genes with GCR ≥ 0.005 (Fig. 4D–F). For P/LP variants, Ashkenazi Jewish and Finnish ethnic groups have the highest risk for intra-ancestry couples (35 and 39 out of 10,000 couples; Fig. 4D). For LoF variants in “known lethal genes”, African/African American population had the highest risk for intra-ancestry couples (78/10,000 couples; Fig. 4E). In the “candidate lethal genes” the highest risk was calculated for the East Asian ethnic group (3,954/10,000 couples; Fig. 4F). Risk values for inter-ancestry couples ranged from 3 to 55 per 10,000 couples for LoF variants in the “known lethal genes” (Fig. 4E), and from 348 to 3,005 per 10,000 couples for “candidate lethal genes” (Fig. 4F).

We also calculated the probability of an individual to carry P/LP or LoF variants in two or more genes in either “known lethal genes” or “candidate lethal genes”. In the general population, 0.01% (P/LP variants in “known lethal genes”), 0.04% (LoF variants in “known lethal genes”), and 1.4% (LoF variants in “candidate lethal genes)” of individuals are predicted to have variants in two genes from the same gene category (Supplementary Fig. S3). Combined, up to 2% of individuals may carry two or more potentially pathogenic variants in known and candidate lethal genes, although the probability to carry variants in multiple target genes may depend on ethnic and familial background of an individual.

### Gene ontology analysis

**Fig. 5:**
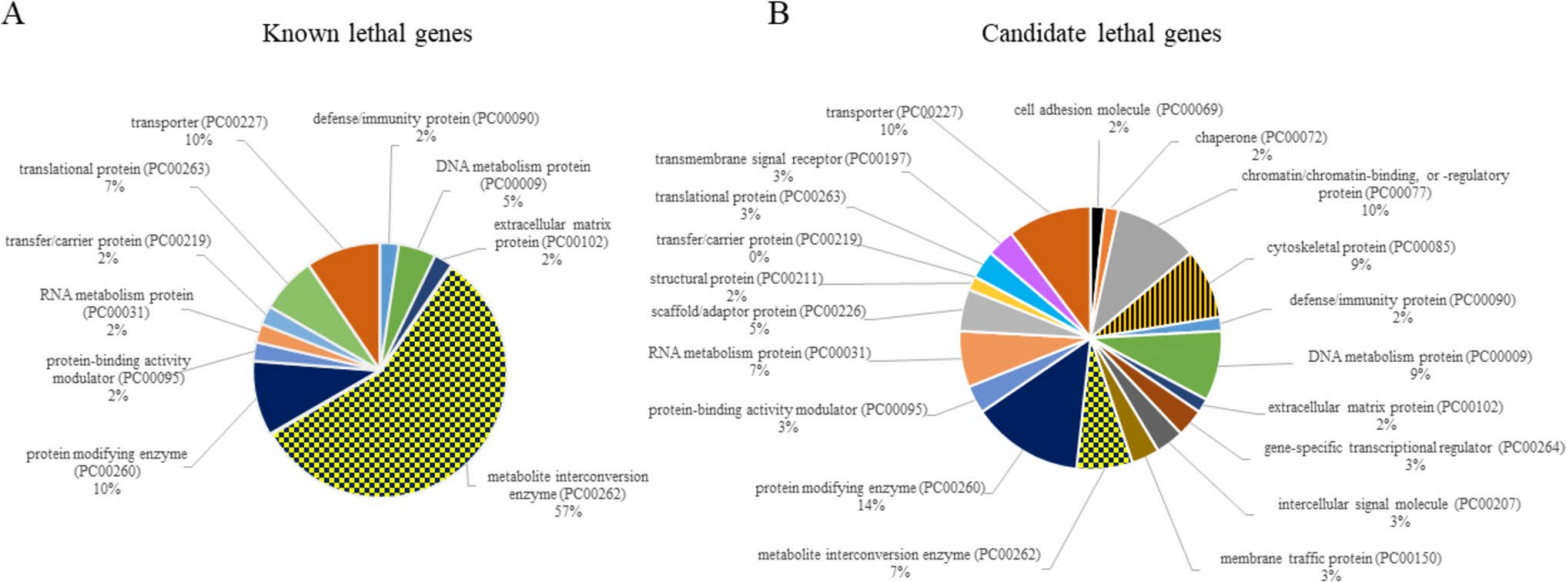
Protein Class Distributions Among Known and Candidate Lethal Genes. This figure utilizes the PANTHER classification system to analyze gene ontology within two categories of lethal genes. Panel **A** illustrates the primary protein classes within known lethal genes. Panel **B** details the primary protein classes within candidate lethal genes, offering insights into the diverse biological functions these genes may influence

Using ontology annotations, comparison between known and candidate gene sets was performed. The “known lethal genes” showed enrichment in metabolic processes. Nearly 70% of entries were genes that code for proteins classified as metabolite interconversion enzyme (PC00262) or protein modifying enzyme (PC00260) (Fig. 5A). In contrast to known lethal genes, candidate lethal genes represent a wide spectrum of proteins (Fig. 5B) associated with developmental processes, regulation, multicellular organism processes, as well as metabolic processes. Importantly, deleterious alterations in candidate genes are more likely to disturb early embryo and fetal development, while defects in the known lethal genes are associated with metabolic conditions leading to neonatal and childhood mortality.

## Discussion

In this study, we conducted a population-based analysis to examine the frequencies of allele carriers of P/LP and LoF variants documented in the gnomAD database for the known and candidate lethal genes. Our study has identified 138 genes that may significantly contribute to pregnancy loss attributable to a high frequency (minimum 0.5%) of potentially lethal variants in the general population.

Nearly 70% of all human conceptions, both clinically and biochemically detected, are lost (Larsen et al. 2013; Tise and Byers 2021). Miscarriages can be traumatic and lead to problems with mental health such as depression, anxiety, and post-traumatic stress disorder (Farren et al. 2020; Quenby et al. 2021). These losses can have multiple causes, including environmental factors (Grippo et al. 2018), immunological (Li et al. 2021), hormonal (Kaur and Gupta 2016; Sullivan 2019), anatomic (Gabbai et al. 2018), and maternal (Frick 2021; Laisk et al. 2020; Larsen et al. 2013). At least 50% of first-trimester miscarriages are not associated with chromosomal abnormalities, suggesting a significant single-gene contribution to euploid losses (Hardy and Hardy 2018). The discovery of lethal genes in humans is hampered by lack of large studies on pregnancy losses and difficulties in evaluation of conceptions at earlier developmental stage. Prior bioinformatic research has suggested that deleterious variants in ~600 human genes are associated with perinatal losses, and homozygous LoF alleles for ~3,400 candidate genes result in non-viable offspring in mice (Dawes et al. 2019). Pre-conceptional screening for such genes can identify at-risk couples for pregnancy losses, allow preimplantation genetic testing and potentially improve implantation rates for in vitro fertilization.

### Contribution of known genes to pregnancy loss and childhood mortality

Only ~3.7% of known human genes are currently linked to stillbirth and childhood mortality based on reports of neonatal death, fetal demise, and discrepancy between expected and observed birth rates in OMIM database (Dawes et al. 2019; Eichstadt et al. 2019; Guo and Gregg 2019; Nowaczyk et al. 2006). Our gene ontology analysis revealed that known lethal genes are primarily involved in metabolic processes. Pathogenic variants in these genes cause recognizable syndromes, manifestations of which can be variable at birth and in utero. Couples who are carriers for pathogenic variants in these genes may experience either livebirth of an affected child or miscarriage, however the factors affecting the severity of the phenotype and onset of fetal/neonatal demise are unknown. In the general population, 5,866 variants in the “known lethal genes” have been classified as P/LP based on their association with specific diseases. Among P/LP, ~60% are LoF variants. We discovered additional heterozygous 14,532 LoF variants in the “known lethal genes” within gnomAD database (Fig. 1). These LoF variants have not been observed in a homozygous state nor linked to a specific disease phenotype in humans, and therefore remain unclassified. Homozygous LoF variants in these genes may cause an identifiable postnatal phenotype or lead to early pregnancy loss. The *DHCR7* gene is one such example among known lethal genes. Pathogenic variants in *DHCR7* result in Smith–Lemli–Opitz syndrome [SLOS, MIM *270400], an autosomal recessive disorder caused by impaired cholesterol metabolism and characterized by dysmorphic facial features, multiple congenital anomalies involving skeletal, cardiovascular, respiratory, and genitourinary systems. Based on our findings and previously published data (Daum et al. 2020; Lazarin et al. 2017), carrier frequency of *DHCR7* P/LP variants ranges from 1/40 to 1/100 individuals among different ethnic groups, with the expected incidence of SLOS between 1/1,600 and 1/10,000 individuals, respectively. The observed incidence of newborns with SLOS in each ethnic group is much lower than expected, suggesting that ~80% of affected conceptions do not survive to birth (Nowaczyk et al. 2006). It is likely that compound heterozygous P/LP and LoF conceptions for the *DHCR7* gene are lost before a pregnancy or SLOS phenotype can be recognized. Although *DHCR7* is included in preconception screening, only P/LP variants will be reported. Both partners might be heterozygous for lethal LoF alleles, however their carrier status and the cause of recurrent pregnancy loss may remain undetermined.

Most of the analyzed variants are rare, however, 1.6% of P/LP variants and 0.37% of LoF variants in 18 “known lethal genes” (*ABCC6, BCKDHB, CEP290, DHCR7, F7, GBA, HBB, KIAA0586, KIAA1109, NLRP7, NPC2, PKHD1, PMM2, PYGM, RARS2*, *SBDS, SPEG,* and *SPINK5)* were seen in 1/200 individuals in the general population. In addition, we identified 43 genes that have P/LP and LoF variants with a frequency of 0.5% or greater in ethnic-specific populations (Fig. 3A and B, Table S2). Deleterious variants in these 61 known genes may greatly contribute to pregnancy losses and neonatal lethality.

### Potential contribution of LoF variants in candidate genes to pregnancy loss

To identify novel autosomal recessive genes, we used bioinformatic and mouse gene candidate approach and focused our analysis on LoF variants in human orthologs. We made significant assumptions, considering that, similar to mouse models, the same recessive mode of inheritance will be present in humans. We assumed that LoF variants in the gnomAD database are functional rather than mere polymorphisms. Furthermore, there's an argument that our focus on LoF variants might have led to an underestimation of the carrier rate among candidate genes, as we excluded missense variants whose pathogenicity remains undetermined.

Our analysis of mouse-lethal human orthologs indicates that about 65% (2,385 out of 3,684, as shown in Fig. 1) of human genes have no known link to human pathologies. The remaining, 35% (1,299/3,684) are associated with known human diseases, and P/LP variants in 350/1,299 genes (27%) are linked to human lethal phenotypes. Analysis of human genotypes in gnomAD database indicated that homozygous variants in 2,612 genes (Fig. 1) were not present in healthy individuals. We identified 77 candidate genes with a heterozygote frequency of at least 0.5% in general population (Table S2) which could significantly contribute to lethal phenotypes in humans. High rate of heterozygous LoF variants together with the absence of individuals carrying homozygous variants for these genes is consistent with pre/perinatal lethality. Therefore, those genes are likely essential for early human development and, are the candidate genes for autosomal recessive lethal conditions in humans.

Interestingly, among 3,684 genes we did not identify heterozygous LoF variants in 263 human orthologs (Fig. 1). Because other types of variants in these genes may still cause fetal lethality and were not included in our analysis, it may underestimate an overall contribution of single gene causes to human pregnancy losses.

In our study, ethnicity-specific analysis shows that Finnish, African/African American, and East Asian ethnic groups have a higher rate of conceiving a child with LoF variants in candidate genes (Fig. 4A–C). Further clinical evidence should be accumulated to establish association of candidate genes to human lethal phenotypes in different populations.

### Functional differences between known and candidate genes

A key distinction between known and candidate genes lies in their functions, which, in turn, affects the timing of potential lethality during fetal development. Candidate lethal genes are involved in more global developmental processes (Fig. 5). LoF variants in these “essential” genes may be less tolerable than those in known genes, potentially leading to lethality at earlier embryonic development stages before the emergence of any discernible fetal phenotype. Certain candidate genes exhibit a notably high prevalence (5–10%) of heterozygous LoF variants in the general population, as shown in (Table S2). For example, LoF variants in *DSCAM* (DS cell adhesion molecule) gene are present in 121 out of 1,000 individuals, and LoF variants in *KMT2E* (lysine methyltransferase 2E) gene are present in 113 out of 1,000 individuals in general population. There are also other genes with common LoF variants in general population such as *CACNA1B* (calcium voltage-gated channel subunit alpha1 B) (81/1,000 individuals), *NUP21* (nucleoporin 214) (95/1,000 individuals), and *TDG* (thymine DNA glycosylase) (108/1,000 individuals). These genes are critical for different global developmental processes such as developing nervous system (*CACNA1B* and *DSCAM*), cellular metabolism, transcriptional regulation, and DNA integrity (*NUP214*, *KMT2E*, and *TDG*).

### Oligogenic causes of pregnancy loss

Given the high carrier rates for certain genes, there is a possibility that both partners may be heterozygous for variants in multiple essential genes. The probability of a couple carrying P/LP or lethal LoF variants in two or more genes is ~1.5% in the general population (Supplementary Fig. S3). This may diminish couple’s probability to conceive an unaffected progeny, as there is a higher chance for each pregnancy to be lost due to multiple monogenic causes or oligogenic etiology.

### Current preconception screenings for the conditions associated with human lethality

Our study compiles a roster of relevant known and candidate genes associated with human lethality, which could be significant for carrier screening. We identified 138 genes (Table S2) with a high frequency rate of ≥ 0.5% of damaging variants present in the general population and various ethnic groups. This includes 61 genes with P/LP and LoF variants in a set of known human genes, and 77 candidate genes with LoF variants, seen in all ethnicities. This suggests that a small proportion of genes (4.5%, or 138 out of 3,080) is responsible for most genetically predisposed non-viable conceptions. Only 29/138 (21%) of the identified genes are currently included in the ACMGG proposed recommendations for preconception genetic carrier screening (Gregg et al. 2021). Moreover, utility of preconception carrier screening for patients with pregnancy losses is limited by only reporting P/LP variants. A large fraction of variants incompatible with life will remain unclassified and unlinked to pre/perinatal lethality and pregnancy loss. Accumulation of evidence and further analysis of unclassified variants along with P/LP variants in the setting of lethal phenotypes and reproductive failures are essential to discern variants responsible for fetal lethality and to classify LoF variants based on the ACMGG guideline. Future PGCS panels augmented by genes and variants associated with fetal lethality would be a substantial opportunity to advance public health and reproductive planning.

### Limitations and further directions

We used a set of variants in the known and candidate genes associated with human/mouse lethality based on the existent clinical and experimental evidence from human and mouse studies, as well as the gnomAD database to filter for variants that appear to be the subjects of natural selection. This study has focused on autosomal recessive inheritance that can be transmitted from the parents, carriers of at-risk alleles, and potentially cause lethality in the fetus and identified during preimplantation genetic testing or diagnosed prenatally. We hypothesized that genes associated with lethality in mouse models follow autosomal recessive inheritance patterns in humans. This assumption is not far-fetched given that 840 out of 1,299 (65%) genes with established phenotype are associated with both, autosomal recessive conditions in humans and lethal phenotype in knockout mice models. We excluded autosomal dominant and X-linked conditions from our considerations to account for hereditary conditions that equally affect conceptions of both sexes. This study did not evaluate other contributing factors to pregnancy loss, including maternal factors. It is possible that the heterozygous variants for some genes associated with placental dysfunction, fetoplacental oxygen homeostasis or angiogenesis, are associated with a reduced reproductive fitness or the carrier status itself impacts the ability of an individual to carry pregnancy to term (Dixon et al. 2017; Rai and Regan 2006). For instance, women carrying heterozygous variants in the *F2* gene (coagulation factor II or prothrombin) may develop thrombophilic conditions during pregnancy (RPRGL2 [MIM *614390]), resulting in intrauterine fetal death, irrespectively of genotype in a fetus (Rai and Regan 2006). Our studies indicate that at least 10% of couples may be at risk of conceiving with a lethal condition, potentially leading to miscarriage. Adding genes linked to miscarriage in preconception screening panels could greatly improve the detection of at-risk couples compared to current panels, which identify only 0.17–2.52% of couples based on ancestry (Guo and Gregg 2019), particularly those that are at risk of carrying variants in multiple genes associated with miscarriage.

To build robust clinical evidence, future studies should focus on large, diverse cohorts of patients experiencing pregnancy loss, infertility, fetal demise, stillbirth, and neonatal death. It is crucial to identify numerous unrelated individuals with rare genotypes to confirm the list of potentially lethal variants and discover new associations between genes and phenotypes. Creating a public database of the “intolerome”—genes and variants that are subjects of biological intolerance and natural selection—is necessary. This database would compile genomic findings, patient phenotypes, medical histories, and reproductive outcomes, aiding future gene discovery and the precise classification of lethal variants and their impact during human development.

### Supplementary Information

Below is the link to the electronic supplementary material.

Supplementary file1 (XLSX 34 KB)

Supplementary file2 (PDF 582 KB)

## Data Availability

The data underlying this article will be shared on reasonable request to the corresponding author.
