# Supplementary material for: Miscarriage risk assessment: a bioinformatic approach to identifying candidate lethal genes and variants
Source: Hum Genet. 2024 Feb 1;143(2):185–95. doi: 10.1007/s00439-023-02637-y (PMC10881709; doi:10.1007/s00439-023-02637-y)
Supplement: Supplementary file 2 — Supplementary file2 (PDF 582 KB) [file 439_2023_2637_MOESM2_ESM.pdf]

SUPPLEMENTARY DATA

GnomAD variant filtration

The entire set of P/LP and LoF variants was filtered out to exclude: (1) loci with less than 50K confidently measured alleles, (2) alleles that are seen in a heterozygous state in ≥5% of individuals from the general or ethnic population, (3) alleles that are seen in a homozygous state in ≥2, (4) low-quality LoF flag variants, (5) LoF variants flagged as problematic by gnomAD, including sites falling in low complexity (lcr), decoy (decoy) and segmental duplication (segdup) regions, (6) counts from the non-canonical transcripts; (7) duplicated entries.

GnomAD v2.1.1 ancestral diversity

Table S1. Populations represented in the GnomAD v2.1.1 exome dataset.

Population	Exomes
African/African American	8,128
Latino/Admixed American	17,296
Ashkenazi Jewish	5,040
East Asian	9,197
Finnish	10,824
Non-Finnish European	56,885
South Asian	15,308
Other	3,070
**Total**	**125,748**

Population risk probability metrics

Variant carrier rate (VCR), frequency of a an individual qualified variant in the general population; gene carrier rate (GCR), a cumulative frequency of all qualified variants in an individual gene; and at-risk couples rate (ACR), the proportion of couples where both partners carry a likely perinatal lethal variant in the same gene were computed as previously described by Guo and Gregg, (doi: 10.1038/s41436-019-0472-7)

Variant Carrier Rate (VCR)

$$VCR = \frac{AC - Hom}{0.5 \times AN}$$

Gene Carrier Rate (GCR)

$$GCR_g = 1 - \prod_{i=1}^{v}(1 - VCR_i)$$

At-risk Couples Rate (ACR)

$$ACR = \sum_{i=1}^{s} GCR_{i,a1} \times GCR_{i,a2}$$

These metrics are given in equations, where AN is the total number of alleles analyzed for the variant, AC is the alternate allele count for the variant, Hom is the number of individuals who are homozygous for the alternate allele; for the GCR of each gene g, the VCRi is the variant carrier rate for variant i, with the cumulative product over v variant targets; for ACR the GCRi,a1 is the GCR for gene i in ancestry-1 and GCRi,a2 is the GCR for gene i in ancestry-2.

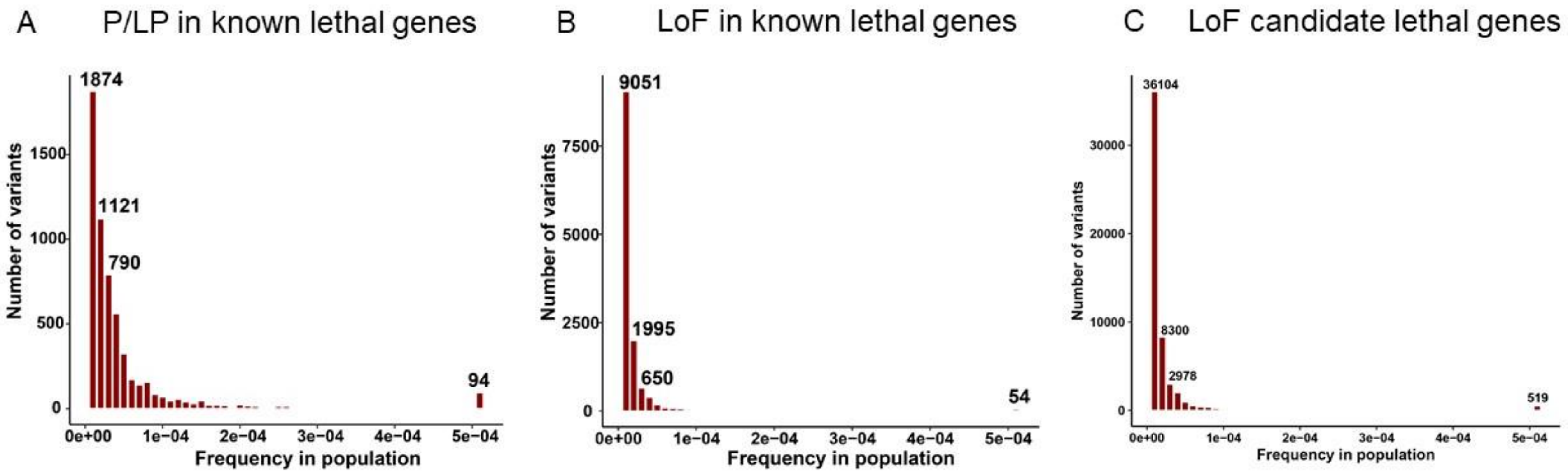

Supplementary Figure S1. Frequency of qualified variants in the known and candidate lethal genes in the general population.

(**A**) VCR of P/LP variants in the known lethal genes. Note, 94 P/LP variants are present with 0.005 allele frequency or seen in 0.5% of population. (**B**) VCR of LoF variants in the known lethal genes. 54 LoF variants are present in 0.5% of population. (**C**) VCR of LoF variants in candidate lethal genes. 519 LoF variants are present in 0.5% of population.

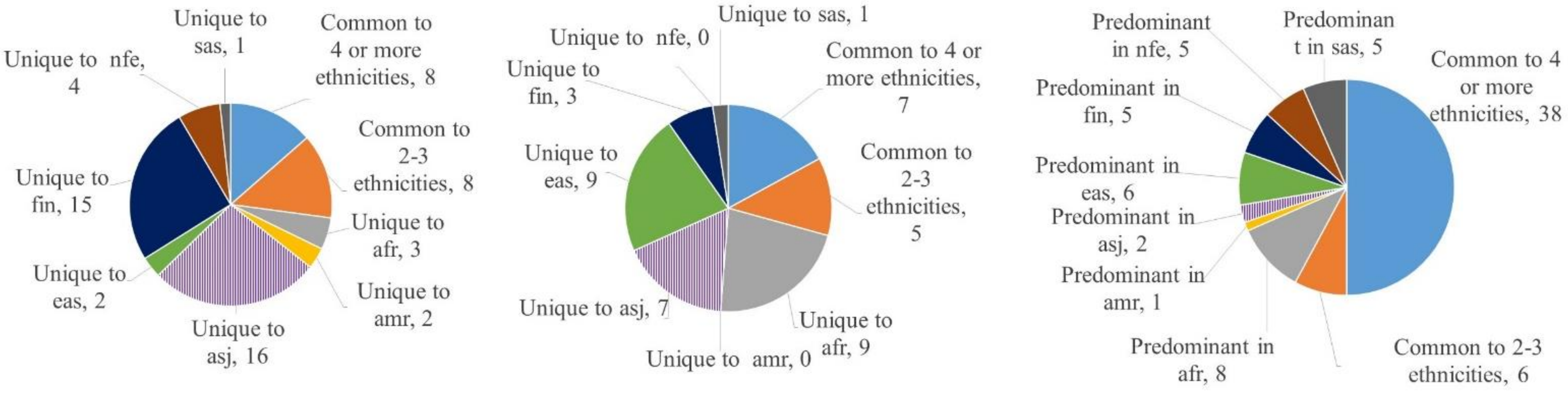

Supplementary Figure S2. Pan-ethnic and population-biased variants.
Common and unique variants in different ethnic groups for (A) P/LP variants in the known lethal genes, (B) LoF variants in the known lethal genes, and (C) LoF variants in candidate lethal genes.

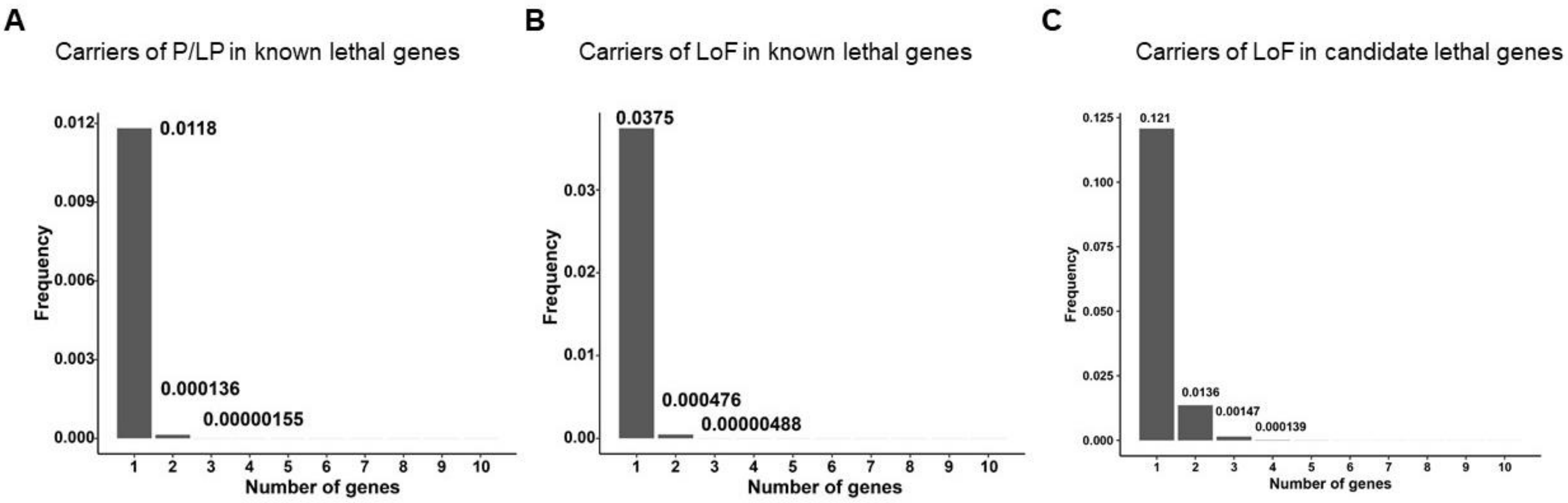

Supplementary Figure S3. Carrier rates for multiple target genes.
Probability for an individual to carry one, two, or several variants when screening for (**A**) P/LP variants or (**B**) LoF variants in the known lethal genes, or for (**C**) LoF variants in the candidate lethal genes.
